# The role of Fibrinogen-like proteins in Cancer

**DOI:** 10.7150/ijbs.56748

**Published:** 2021-03-08

**Authors:** Jing Yu, Jing Li, Jing Shen, Fukuan Du, Xu Wu, Mingxing Li, Yu Chen, Chi Hin Cho, Xiaobing Li, Zhangang Xiao, Yueshui Zhao

**Affiliations:** 1 Laboratory of Molecular Pharmacology, Department of Pharmacology, School of Pharmacy, Southwest Medical University, Luzhou, Sichuan, China.; 2 South Sichuan Institute of Translational Medicine, Luzhou, Sichuan, China.; 3 Department of Oncology and Hematology, Hospital (T.C.M) Affiliated to Southwest Medical University, Luzhou, Sichuan, China.; 4 Department of Pharmacy, The Affiliated Hospital of Southwest Medical University, Luzhou, Sichuan, China.

**Keywords:** FGF1, FGF2, Cancer

## Abstract

Fibrinogen-associated protein (FREP) family is a family of proteins with a fibrin domain at the carboxyl terminus. Recent investigations illustrated that two members of FREP family, fibrinogen-like protein-1 (FGL1) and fibrinogen-like protein-2 (FGL2), play crucial roles in cancer by regulating the proliferation, invasion, and migration of tumor cells, or regulating the functions of immune cells in tumor microenvironment. Meanwhile, they are potential targets for medical intervention of tumor development. In this review, we discussed the structure, and the roles of FGL1 and FGL2 in tumors, especially the roles in regulating immune cell functions.

## Introduction

Fibrinogen is a glycoprotein composed of the central nodule, coiled-coil domain, and α, β, and γ C-terminus domains 1. The carboxyl-terminal β and γ domains form a 30 kD globular field, which showed highly homologous with fibrinogen-like globe (FBG) domains in other kinds of proteins 2. The evolutionarily conserved proteins with FBG structures are called fibrinogen-related proteins (FREPs), which are universally expressed in mammals and invertebrates 3, 4. The multiplicity of carboxyl-terminal subdomains of FREPs demonstrate the comprehensive functional diversity, from cell signaling to pathogen binding to fibrin gel formation 5. In the past decade, several FREPs mainly have been played a battery of roles in various species. For example, experimental certification for FREPs with bacteriolytic activity in vertebrates 6, phenomenon of innate immunity, peculiarly in the identification of pathogens 3, 7-9, varying parts during wound healing 2. Besides, FREPs have been exhibited to resist infection 10 and stable bacterial agglutinating activity 11.

A growing body of investigations highlights that members of FREP superfamily, such as fibrinogen-like protein-1 (FGL1), and fibrinogen-like protein-2 (FGL2), play pivotal roles in cancer and in modulating immune cell functions. FGL1, also named as hepatocyte-derived fibrinogen-like protein-1 (HFREP1) 11, comprises a hepatocyte-secreted protein that was originally cloned in human hepatocellular carcinoma (HCC) 12-14 and conduces to mitogenic and metabolic activity 15. FGL1 is inextricably linked to obesity 16. Meanwhile recent researches have highlighted that FGL1 may be a dramatic and important benchmark for measuring radiation-induced liver damage 17. FGL1 serves as a pivotal “bridge” between liver regeneration and adipose tissue function 18. FGL1 is induced to an acute phase reactive reaction by interleukin- 6 (IL-6) 19. Furthermore, FGL1 plays important roles in cancers, and is a potential target for cancer treatment.

FGL2 is a 439 AA protein with 36% homology in β and γ chains of fibrinogen 20, and was initially cloned from cytotoxic T lymphocytes 21. FGL2 shows prothrombin enzyme activity and plays immunomodulatory function in a diversity of diseases, including viral-induced inflammation, xenograft rejection, chronic obstructive pulmonary disease, autoimmune disorders 22, 23, abortion and tumor growth 24-27. FGL2 is principally generated by tumor cells, activated macrophages, T cells, and endothelial cells 28-31.

In this review, we overview the current understandings of the roles of FGL1 and FGL2 in cancer microenvironment, and review the roles of FGL1 and FGL2 as potential immunotherapeutic targets for cancer therapy.

## Structure and Function

### FGL1

FGL1 possesses a fibrinogen-related domain in its C-terminal proportion, whereas three functional domains of platelet binding site, crosslinking region, and thrombin-sensitive site are not contained 13, 19. FGL1 constitutes an N-terminal coil-coil domain and a C-terminal fibrinogen-like domain 32, which is formed by two disulfide bonds connected by a 34 kD homologous dimer 19, 33 and an N-terminal signal recognition peptide 18. FGL1 facilitates fine-tune systemic inflammation by allowing feasible cross-talk between the liver and other peripheral tissues under normal physiological conditions, nevertheless some solid tumors will break this state, they will enhance the expression of FGL1 15. FGL1 plays key role in liver regeneration and the suppression of hepatocyte apoptosis with the help of bone marrow-derived mesenchymal stem cells (BMSCs) 34. FGL1 is highly generated by human cancer cells, and elevation of plasma levels of FGL1 in tumor patients is associated with resistance to PD-1/PD-L1 therapy 15. While a study showed that FGL1 promotes HCC tumor formation by inhibiting the activation of antigen-specific T cells 15, another study found that FGL1 could inhibit the growth and proliferation of HCC, which runs counter to previous conclusions 19. Therefore, the actual effect of FGL1 on hepatocytes is controversial 17.

### FGL2

**Figure 1:**
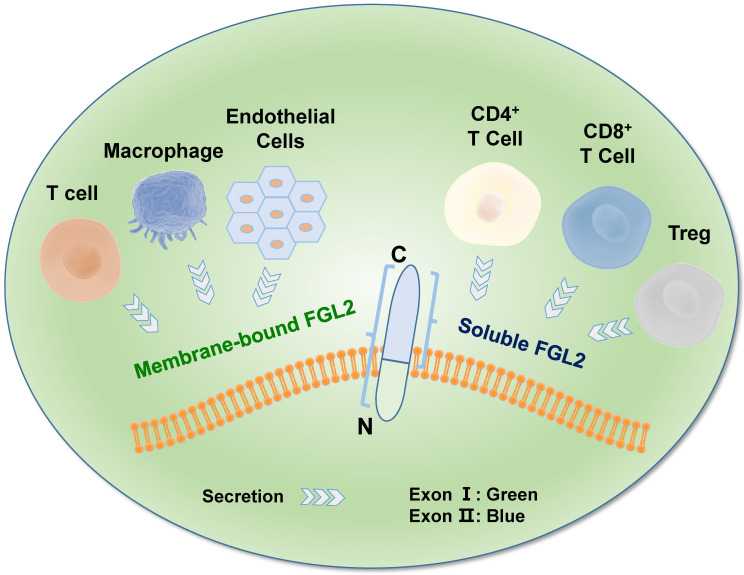
** Molecular structure of FGL2.** The *FGL2* gene contains two exons, which were separated by an intron. The mFGL2 contains the membrane, transmembrane, and extramembrane regions, while sFGL2 only has the extracellular domain. The amino terminal in the *FGL2* gene structure is located inside of the membrane, and the carboxyl terminal is located outside of the membrane. mFGL2 is mainly secreted by macrophages, endothelial cells, and T cells, while sFGL2 is secreted by CD4^+^ T cells, CD8^+^ T cells, and Tregs.

FGL2, also known as FGL2-prothrombin, is a 64-70 kD type 2 transmembrane protein with 439 amino acids (AA), which shows 36% homologous to the β and γ fibrinogen chains 35. It is predicted that the length of 70-kD protein is 439 amino acids (AA), with the N-terminus containing a 2 AA-long cytoplasmic domain and a 21-AA-long transmembrane domain. The remaining 416 AA constitutes the extracellular domain 36 in the *FGL2* protein structure. The *FGL2* gene possesses two exons, exon I encodes the first 204 amino acids, and exon II encodes the remaining 234 amino acids (**Figure 1**) 37. Two diverse forms of FGL2 protein, membrane bound FGL2 (mFGL2) and soluble FGL2 (sFGL2), were characterized 38. mFGL2 were assembled by three domains, the membrane, transmembrane, and extramembrane domains, whereas sFGL2 only maintains the extracellular domain and is secreted into plasma 28.

FGL2 can directly cleave prothrombin into thrombin without factor VII or factor X 36, 39, leading to cascade reaction 40. FGL2 is an immunomodulator that plays an instrumental role in innate immunity. A growing body of evidence indicated that FGL2 serves as a T regulatory (Treg) effector by inhibiting T cell activity in a FoxP3-dependent manner 41-43. Its formidable supervisory system is manifested in the role of the adaptive immune system, and it has been indicated that combining FcγRIIB with FGL2 can prevent the maturation of dendritic cells (DCs) 21, 44 and B cell function^45^, and can cause FcγRIIB-mediated-CD8^+^ T cells apoptosis 46. Moreover, FGL2 also involves in tumor development 36, and has been perceived to the overexpression of FGL2 in tumor and interstitial inflammatory cells 29. The tumor section staining displayed that the expression of FGL2 was raised in most CD57^+^, CD68^+^, CD8^+^ T cells, and vascular endothelial cells 29. The latest study by Vavougios et al. showed that FGL2 is associated with SARS-CoV-2 infection, but the detail mechanism still unclear 47.

#### mFGL2

mFGL2, a type II transmembrane glycoprotein protein overexpressed in many types of tumors, and possesses serine protease activity on the surface of macrophages, T cells, and endothelial cells 30. The N-terminal linear coiled-coil domain of mFGL2 is answerable for its prothrombinase activity 48. mFGL2 can directly cleave prothrombin into thrombin without factor VII or factor X 36, 39, leading to cascade reaction 40.

#### sFGL2

**Figure 2:**
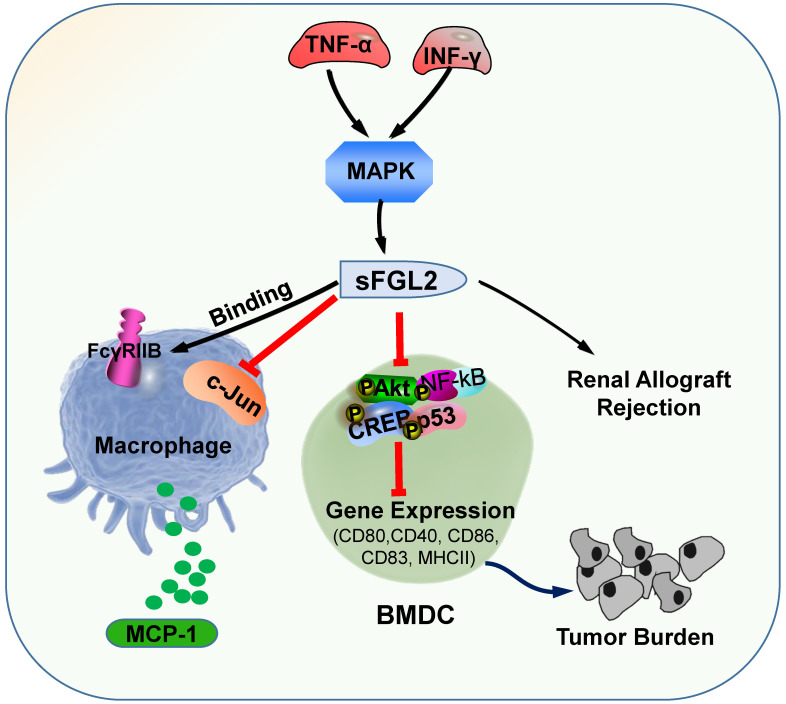
** The primary roles of sFGL2.** sFGL2 inhibited macrophages by binding with FcγRIIB receptor to release MCP-1 to attenuate c-Jun N-terminal kinase activation. TNF-α and IFN-γ motivate sFGL2 through MAPK signaling to promote renal allograft rejection. sFGL2 inhibits the expression of MHCII, CD40, CD80, CD86 and CD83 and phosphorylation of Akt, NFκB, cAMP response element binding protein (CREB) and p38 in BMDC and reinforce the burden of tumor.

sFGL2, an oligomer composing of four disulfide-linked FGL2 monomers, lacking the N-terminal hydrophobic sequence, only contains the C-terminal domain, which is reliable for the immune regulatory effect. sFGL2 is generally secreted by CD4^+^, CD8^+^ T cells, and Tregs 49. It has been demonstrated that sFGL2 could suppress the proliferation of T cell, maintain the immunosuppressive activity of Tregs, and prohibit the maturation of DCs 21, 50. The discrepancy between mFGL2 and sFGL2 in the tertiary structure contributes to the difference in function 29. In renal allograft rejection, certain concentrations of TNF-α and IFN-γ can stimulate CD4^+^ T cells to secrete sFGL2 through MAPK signaling 51. sFGL2 moderated acute rejection (AR) by inducing Kupffer cells(KCs) M2 polarization 52. Previous studies documented the role of sFGL2 in malignancies and autoimmune diseases, whereas recent studies have found that it also plays crucial role in the immunotherapy efficiency for malaria parasites treatment 53. sFGL2 inhibited macrophages by binding to FcγRIIB receptor to block the release of monocyte chemoattractant protein-1 (MCP-1), and by suppressing the activation of c-Jun N-terminal kinase 53. sFGL2 can restrain the expression of MHCII, CD40, CD80, CD86, and CD83 in bone marrow derived mesenchymal stem cells *in vitro* by inhibiting the phosphorylation of Akt, NFκB, cAMP response element binding protein (CREB), and p38 in DC, and can decrease the cytotoxicity in HCC tissues and reinforce the burden of tumor 54. sFGL2 levels were detected for the first time in the Egyptian HCV-infected and HCC patients, which provided a potential immune target for the treatment of HCV and HCC in the future (**Figure 2**) 55.

## FGL1 and FGL2 Signaling Pathways

### FGL1

**Figure 3:**
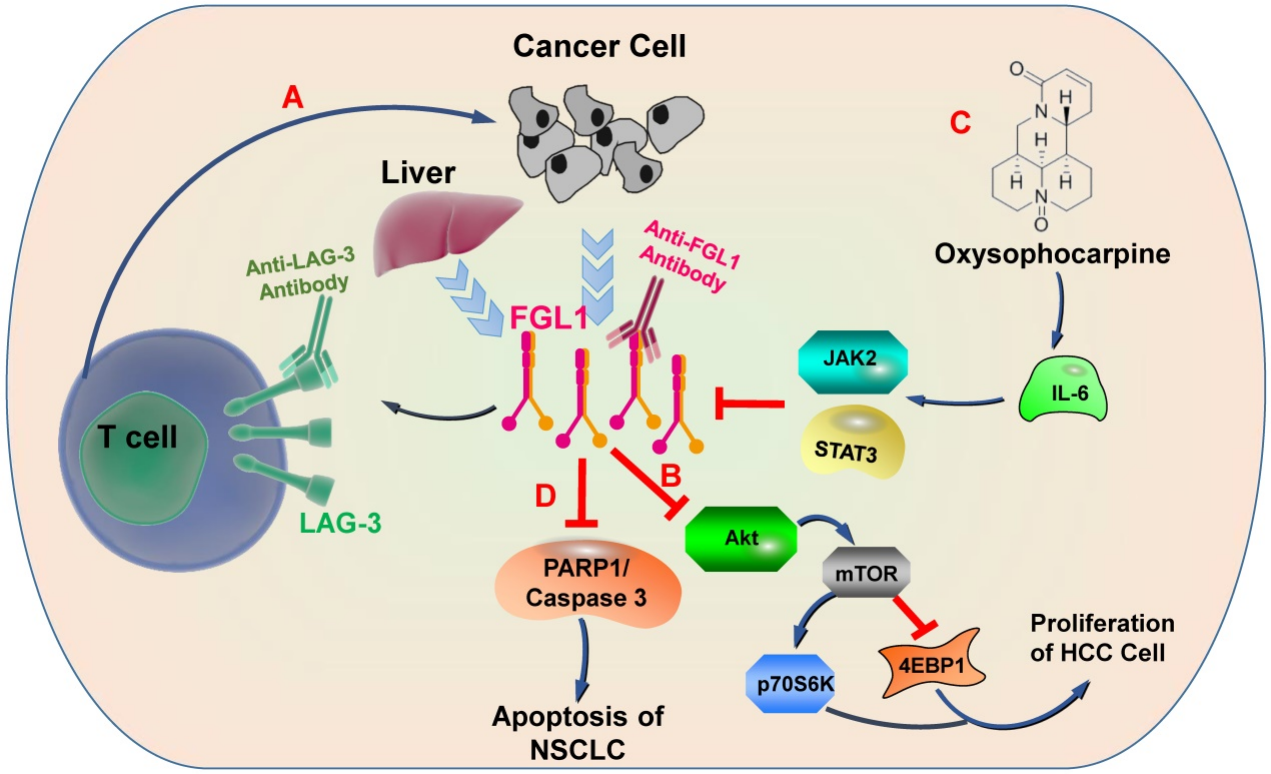
** FGL1-involved signaling pathways.** A) FGL1 is normally released by the liver in low levels but by cancer in high levels. FGL1 is identified as a major ligand for the inhibitory receptor LAG-3, and its blockade can potentiate anti-tumor T cell responses. B) FGL1 inhibits liver cancer cell proliferation by suppressing Akt signaling. C) The expression of FGL1 is down-regulated through Oxysophocarpine, which suppresses IL-6-mediated JAK2/STAT3 signal activation and subsequently strengthen the effects of anti-LAG-3 immunotherapy. D) FGL1 promotes NSCLC by regulating the PARP1/ Caspase 3 pathway.

Physiologically, FGL1 promotes the proliferation of human normal hepatocytes by motivating the EGFR/ERK cascade through the Src-dependent mechanism 56. FGL1 induces insulin resistance through an ERK1/2-dependent pathway in hepatocytes, and induces adipogenesis by stimulating the ERK1/2-C/EBPβ-dependent pathway 16. Some non-small cell lung cancer (NSCLC) patients are acquired resistance to gefitinib, a drug for NSCLC therapy, Sun et al. found that FGL1 was upregulated in gefitinib-resistant cancer cells, and mediated the resistance to gefitinib in NSCLC cells by regulating the PARP1/ Caspase 3 pathway 57. Lymphocyte activation gene 3 (LAG-3) is a transmembrane protein that is generally expressed on the surface of activated immune cells, such as NK cells, T cells, and other immune cells 58. Its major function is to act as a receptor to transmit inhibitory signals and negatively regulate the function of CD4^+^ and CD8^+^ T cells 59. Current study has displayed that FGL1 is the considerable ligand of the LAG-3 and FGL1-LAG-3 pathway contributes to tumor growth 15. Deviant activation of numerous signaling pathways is an essential characteristic of HCC 60. The expression of FGL1 is down-regulated by oxysophocarpine, which restrains IL-6-mediated JAK2/STAT3 signal activation and subsequently strengthens anti-LAG-3 immunotherapy 61. Nayeb-Hashemi et al. demonstrated that FGL1 mainly regulates Akt signaling pathway in HCC, knockout of FGL1 enhanced the activation of Akt compared with wildtype FGL1 control group 33. Meanwhile, knockout of FGL1 also increased the phosphorylation of 4EPB1 and p70S6K, which are targets of mTOR (**Figure 3**) 33.

### FGL2

As an immune regulator, FGL2 is involved in various signaling pathways in inflammatory diseases and tumors. Recent studies have shown that FGL2 plays a crucial role in the pathogenesis of experimental and human fulminant and chronic viral hepatitis 62. Li et al. illustrated that FGL2 blockades NF-κB signaling to attenuate DSS-induced inflammatory bowel disease (IBD) 63. Some studies measured the protein expression levels of NF-κB signal related factors, such as p-IKKα/β, IKKα/β, p-IκBα, IκBα, p-p65 and p65, and NF-κB signal transduction downstream proteins iNOS, Cox-2 and TNF-α by western blotting in DSS-induced IBD, the results of the study demonstrated that compared with wild-type mice, the expression levels of these proteins in FGL2-knockout mice were increased 64. Xu et al. found that the increase of FGL2 in viral fulminant hepatitis (FH) from MHV-3 infection relies on the participation of complement component 5 (C5) and its receptor C5R 65. The C5a/C5aR signaling participates in MHV-3 induced FGL2 expression by activating kinases of ERK1/2 and p38 MAPK 65. Moreover, another study by McGilvray et al. also demonstrated that MHV-3 induces the expression of macrophage prothrombin FGL-2 through p38 MAPK pathway 66. Liu et el. illustrated that the regulation of FGL2 transcription was regulated by the STAT1-dependent pathway and Sp1/Sp3-STAT1/PU.1 transcriptional complex 67. Jia et al. found that TNF-α induced-endothelial cell FGL2 improves microcirculation dysfunction through NF-κB and P38 MAPK signaling pathways 68. In another study, it was also confirmed that FGL2 activates this pathway and the NLRP3 inflammasome aggravated nonalcoholic steatohepatitis (NASH) and promoted lipid metabolism disorders 69. These studies fully verified that FGL2 mediated by NF-κB and MAPK signaling pathways can accelerate the development of inflammation. IL-1 alone is incapable on regulating FGL2 expression, but synergistic TNF-α can enhance the expression of FGL2 through the NF-κB pathway 70. Han et al. examined the transcription of the hFGL2 gene in response to hepatitis B core (HBc) and hepatitis B virus X protein (HBx) proteins ERK promotes hBC-induced nuclear C-est-2 DNA binding activity and FGL2 induction, while JNK promotes HBX-induced nuclear C-est-2 DNA binding activity and FGL2 induction, ERK and JNK belong to the three core elements of MAPK pathway, thereby MAPK signaling pathway is involved in FGL2 transcription 62. A recent study showed that the deficiency of FGL2 in a mouse model aggravated UUO-induced renal fibrosis by upregulation STAT6-dependent M2 macrophage polarization 71.

**Figure 4:**
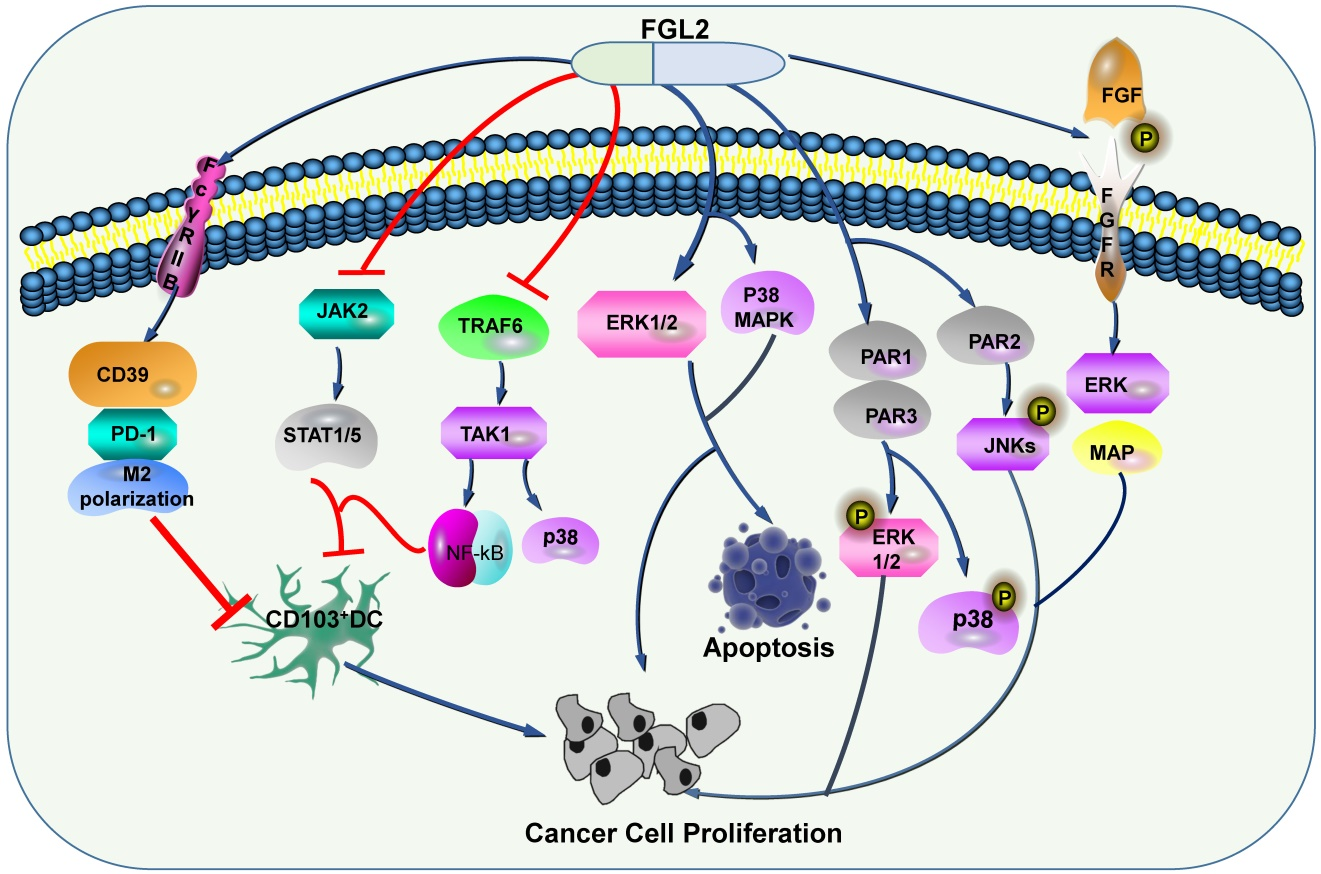
** FGL2-related signaling pathways in tumors.** By regulating a series of signaling pathways, FGL2 promotes the apoptosis of tumor cells, and inhibits dendritic cells to promote tumor progression.

Some researchers discussed that FGL2 can induce the expression levels of CD39 and PD-1, and induce M2 polarization of macrophage 72, and the apoptosis of DC through FcγRIIB pathway 45. Apart from FGL2 blocks TRAF6/TAK1/NF-κB/p38 signal and JAK2/STAT1/5 in response to GM-CSF, thereby restraining CD103 induction on DCs, thus stimulates the growth of tumor cells 73. In HCC, Liu et al. proposed that FGL2 associated with tumor cells is produced by thrombin and promotes tumor proliferation *in vitro* and tumor growth *in vivo*. These phenomena have gone through two signaling pathways. One is the production of FGL2, which can activate PAR1 and PAR3 to cause phosphorylation of ERK and P38, and the other is the production of FGL2, which can activate PAR2 and cause JNK phosphorylation 74. Ming Tang and his colleagues illustrated that FGL2 expression in fresh clear cell renal cell carcinoma (ccRCC) tissues was conspicuously up-regulated, furthermore, they confirmed that high expression level of FGL2 blockades ccRCC cell viability, activates ERK1/2 and p38 MAPK pathways, and promotes apoptosis (**Figure 4**) 75.

## The role of FGL1 and FGL2 in tumors

### FGL1

Gene expression analyses indicated that the expression levels of FGL1 were increased in human solid tumors, including colorectal cancer, prostate cancer, melanoma, lung cancer, and breast cancer, especially in lung adenocarcinoma, while were decreased in head and neck cancer, pancreatic cancer, and liver cancer compared with normal tissues, based on the data from the BioGPS tissue microarray database and the Cancer Genome Atlas (TCGA) database 15. Analysis of gastric cancer (GC) data collected from ATGC illustrated that contrast to normal gastric tissue, FGL1 expression is upregulated in GC, and was associated with poor prognosis 12. Meanwhile, FGL1 accelerates the proliferation, migration, and invasion of GC cells, accordingly FGL1 can be used as a target for the treatment of GC and a predictor of the prognosis of GC patients 12. Gefitinib resistance can be regulated by FGL1 through inhibiting the apoptosis of the non-small cell lung cancer (NSCLC) cell line PC9/GR, and FGL1 can act as a latent therapeutic target for NSCLC 57. Besides, Chen et al. have conducted in-depth studies on the role of FGL1 that they inoculated MC38 (a type of colon cancer cell) through mice and found that the tumor growth of FGL1-KO mice was significantly slower than that of WT mice 15.

It is of interest that FGL1 owns both pro-tumor and anti-tumor functions. Bie et al. pointed out that FGL1 expression increased in LKB1 mutant lung adenocarcinoma through functional experiments and bioinformatics data analysis, and it can ameliorate epithelial-mesenchymal transformation (EMT) and angiogenesis in LKB1 mutant lung adenocarcinoma 76. Hamed Nayeb-Hashemi et al. indicated that FGL1 serves as a tumor suppressor in HCC, knockout FGL1 expression strengthens the Akt/mTOR signaling pathway, thus supporting FGL1 as a therapeutic target for HCC 33.

### FGL2

Some studies have demonstrated that FGL2 is expressed in human gliomas, and its expression is involved in the malignant transformation from low-grade gliomas (LGGs) to high-grade gliomas (HGGs), which indicates that the expression level of FGL2 is related to the grade of gliomas 72. FGL2 conduces to GBM progression though stimulating immunosuppression mechanisms 41, 72. Latha et al. knocked out FGL2 in GL261 cells, and constructed stable cell lines, which were injected into mice. Compared with mice injected with GL261 cells alone, the former had no tumor formation at all. These phenomena suggest that FGL2 is an indispensable factor in the formation of invasive tumors in GBM 41. Meanwhile, FGL2 could promote the polarization of macrophages and the proliferation of Treg cells in tumor microenvironment, thus enhancing the immunosuppressive function 41. FGL2 which derived from glioma cells acts as an immunosuppressive manipulator though up-regulating the expression levels of CD39 and PD-1 72, and restraining the differentiation of CD103^+^ DCs 73. In GBM, targeting expression of FGL2 *in vivo* can strengthen the immune function and improve the therapeutic outcome of glioma patients 77. *In vitro* studies have shown that knockdown of FGL2 expression can inhibit the proliferation of HCC cells by arresting G0/G1 cell cycle and affecting angiogenesis in HCC 78, or by promoting the accumulation of myeloid-derived suppressor cells (MDSCs), which promote cancer progression, in liver tumor microenvironment 79. Studies have shown that Ad-hFGL2-miRNA (an adenoviral vector expressing anti-hFGL2 artificial miRNA) proscribes tumor growth and is involved in its repression of hFGL2 and attenuation of angiogenesis. In addition, in the established orthotopic liver cancer model, its anti-tumor efficacy was further verified 78. The proangiogenic/protumorigenic activities of FGL-2 is mediated by FGF/ERK signaling, which combines with its receptor FGFR and results in FGFR autophosphorylation and activation, and eventually signal transduction via multiple downstream pathways, containing ERK/MAP kinase (**Figure 4**) 36 . In the gene-array experiments using PC-3 (a wild type human prostate carcinoma cell line) clones, silencing FGL2 remarkable downregulates FGF-2, thus inhibiting the occurrence of prostate cancer in mice 36. Stromal derived FGL2 facilitates the growth of lung cancer by imitating the tumor-promoting microenvironment instead of regulating tumor cells directly. Zhu and his team indicated that knockdown FGL2 in mice slugged the progression of lung cancer, inhibited CXCL12-mediated cumulation of MDSCs in TME, and weakened the role of CAFs 80. Some studies have demonstrated that FGL2 promotes the proliferation of CRC both *in vitro* and *in vivo* 81. Furthermore, FGL2 prothrombinase could contribute to tumor hypercoagulability and presumably to angiogenesis 29. In the study of prothrombin activity of FGL2 in peripheral blood monocytes of B-cell lymphoma, Rabizad et al. measured the thrombin level of non-Hodgkinundefineds lymphoma (NHL) and normal controls and indicated that the activity of FGL2 prothrombin in NHL is elevated in active lymphoma, which can be used as a prospective marker for remission of lymphoma 39.

Interestingly, Yuan et al. utilized TIMER database analysis to reveal that FGL2 was positively correlated with infiltration of immune cells such as DC, macrophages, B cells, and CD8^+^ and CD4^+^ T cells in lung adenocarcinoma and exerts anti-tumor activities 82. FGL2 probably serves as a beneficial marker for the treatment of lung adenocarcinoma. In addition, Feng et al. revealed that the expression of FGL2 in breast cancer was also significantly decreased, and the expression of FGL2 was positively correlated with anti-tumor immune cells such as B cells, T cells, macrophages and DC in breast cancer 83. Strikingly, in another study, it was also verified that the deficiency of FGL2 can accelerate colitis-associated colorectal cancer (CAC) evolution 84. Additionally, Liu et al. utilized MAPK inhibitors to significantly reduce the expression level of FGL2 in CAC cells and FGL2 ameliorated the invasion and migration of CRC cells 85. Moreover, in CAC, patients with FGL2 overexpression have a poor prognosis overall, and FGL2 overexpression probably accelerates tumor progression by inducing epithelial-macrophage transformation.

## Targeted therapy and immunotherapy

Blockading the interaction of FGL1 and LAG-3 can trigger T cells and restore anti-tumor immunity by facilitating the TCR/CD28 signaling pathway 86. FGL1 silencing accelerates CD8^+^ and CD4^+^ T cell immunity against tumor growth 15. FGL1 accelerates anti-LAG-3 immunotherapy under the mediation of oxysophocarpine 61.

The immunomodulatory function of FGL2 has been a hot topic. FGL2 binds to the receptors on antigen presenting cells (APCs) to exert its immune regulatory activity 45. Yan et al. established stable FGL2KO tumor cell lines by CRISPR/Cas9 technology, and implanted FGL2KO tumor cells and Ctrl cells into mice. The results displayed that the anti-tumor effect of CD8^+^ T cells was enhanced in FGL2KO mice. Moreover, they demonstrated *in vitro* that FGL2 can prohibit the development of CD103^+^ DC induced by GM-CSF, and then T cells were not motivated, thereby leading to the occurrence of GBM 73. Studies have indicated that FGL2 was positively correlated with several immunosuppressive mediators including PD-1, PD-L2, CD39, BTLA, LAG-3, IL-10 and TGFβ1 though applying Pearson's correlation analysis. In addition, whether it is the original brain tumor mouse model or the mouse brain infiltrating lymphocytes inoculated with GL261-FGL2, compared with the control model, the number of MDSC, M2 and CD39^+^Treg increased 72, indicating that FGL2 reinforces tumor immunosuppression.

Fgl2 ^+^/^+^ mice and FGL2^-^/^-^ mice in intravenous infection of lymphocytic chori-omeningitis virus clone-13 (LCMV), Luft and his colleagues suggested the total number of macrophages and DC expressing CD80, CD86, MHCII and virus-specific CD8^+^ T cells were significantly enhanced in fgl2^-^/^-^ mice in contrast to fgl2^+^/^+^ mice. At the same time, the percentage of CD4^+^ and CD8^+^T cells expressing PD-1 decreased. Inhibition of the FGL2 promotes antiviral T-cell and B-cell responses 87. Recent studies have showed that exogenous FGL2 inhibits the cytotoxicity of NK cells 88.

## Conclusions and Perspectives

In the past few years, a series of emerging evidences have exhibited the immunoregulatory effects of FGL1 and FGL2 as new effector molecules. Currently, researches on FGL1 are still lacking, which principally studies its basic role in tumor and immunity. In the present studies, we acquaint that FGL1 promotes the development of GC, NSCLC and CRC, but inhibits the expansion of these tumors in LKB1 mutant lung adenocarcinoma and HCC, which is consistent with the previous ATCC data analysis. In addition to these, the immune function of FGL1 is mainly reflected in its combination with LAG-3, which weakens the cytotoxicity of CD8^+^ T cells and contributes to tumor growth. FGL2 is expressed in malignant tumor including GBM, HCC, prostate carcinoma, B-cell lymphoma, CRC, CAC, and lung adenocarcinoma from patients. Apart from cancer, FGL2 gene is also conspicuously correlated with infiltrating mesenchymal cells including DCs, macrophages, NK cells, CD8^+^ T lymphocytes and vascular endothelial cell.

Although FGL1 and FGL2 are associated with tumor therapy, they are limited to basic studies and lack of further experimental results and clinical studies reporting that they can be used as biomarkers for diagnosis and prognosis. Therefore, future research should focus not only on the mechanism of its action but also on the clinic application of these proteins. Taken together, further exploration of FGL1 and FGL2 will deepen our understanding of the induction and maintenance of immune tolerance, thus promoting the development of new strategies for the treatment of multiple tumor-related immune diseases.

## Abbreviations

FREP: Fibrinogen-related protein
FGL1: fibrinogen-like protein-1
FGL2: fibrinogen-like protein-2
FBG: fibrinogen-like globe
HFREP1: fibrinogen-related proteins
HCC: hepatocellular carcinoma
IL-6: interleukin- 6
BMSCs: bone marrow-derived mesenchymal stem cells
AA: amino acids
mFGL2: membrane bound FGL2
sFGL2: soluble FGL2
Treg: T regulatory
DCs: dendritic cells
AR: acute rejection
KCs: Kupffer cells
MCP-1: monocyte chemoattractant protein-1
CREB: cAMP response element binding protein
NSCLC: non-small cell lung cancer
LAG-3: lymphocyte activation gene 3
IBD: inflammatory bowel disease
FH: fulminant hepatitis
C5: component 5
NASH: nonalcoholic steatohepatitis
HBc: hepatitis B core
HBx: hepatitis B virus X protein
ccRCC: clear cell renal cell carcinoma
TCGA: the Cancer Genome Atlas
GC: gastric cancer
EMT: epithelial-mesenchymal transformation
LGGs: low-grade gliomas
HGGs: high-grade gliomas
MDSCs: myeloid-derived suppressor cells
NHL: non-Hodgkinundefineds lymphoma
CAC: colitis-associated colorectal cancer
APCs: antigen presenting cells
LCMV: lymphocytic choriomeningitis virus clone-13
